# Improvement of Heart Failure by Human Amniotic Mesenchymal Stromal Cell Transplantation in Rats

**Published:** 2016-07-06

**Authors:** Seyed Mohammad Taghi Razavi Tousi, Mahdieh Faghihi, Maliheh Nobakht, Mohammad Molazem, Elham Kalantari, Amir Darbandi Azar, Nahid Aboutaleb

**Affiliations:** 1 *Department of Physiology, School of Medicine, Tehran University of Medical Sciences, Tehran, Iran.*; 2 *Medical Biotechnology Research Center, Guilan University of Medical Sciences, Rasht, Iran.*; 3 *Physiology Research Center, Iran University of Medical Sciences, Tehran, Iran.*; 4 *Department of Veterinary Diagnostic Imaging, Faculty of Veterinary Medicine, University of Tehran, Tehran, Iran.*; 5 *Oncopathology Research Center, Iran University of Medical Sciences, Tehran, Iran.*; 6 *Rajaie Cardiovascular, Medical and Research Center, Iran University of Medical Sciences, Tehran, Iran.*; 7 *Physiology Research Center, Iran University of Medical Sciences, Tehran, Iran.*

**Keywords:** *Heart failure*, *Mesenchymal stromal cells*, *Cell transplantation*

## Abstract

**Background: ** Recently, stem cells have been considered for the treatment of heart diseases, but no marked improvement has been recorded. This is the first study to examine the functional and histological effects of the transplantation of human amniotic mesenchymal stromal cells (hAMSCs) in rats with heart failure (HF).

**Methods: ** This study was conducted in the years 2014 and 2015. 35 male Wistar rats were randomly assigned into 5 equal experimental groups (7 rats each) as 1- Control 2- Heart Failure (HF) 3- Sham 4- Culture media 5- Stem Cell Transplantation (SCT). Heart failure was induced using 170 mg/kg/d of isoproterenol subcutaneously injection in 4 consecutive days. The failure confirmed by the rat cardiac echocardiography on day 28. In SCT group, 3×10^6^ cells in 150 µl of culture media were transplanted to the myocardium. At the end, echocardiographic and hemodynamic parameters together with histological evaluation were done.

**Results: ** Echocardiography results showed that cardiac ejection fraction in HF group increased from 58/73 ± 9% to 81/25 ± 6/05% in SCT group (p value < 0.001). Fraction shortening in HF group was increased from 27/53 ± 8/58% into 45/55 ± 6/91% in SCT group (p value < 0.001). Furthermore, hAMSCs therapy significantly improved mean diastolic blood pressure, mean arterial pressure, left ventricular systolic pressure, rate pressure product, and left ventricular end-diastolic pressure compared to those in the HF group, with the values reaching the normal levels in the control group. A marked reduction in fibrosis tissue was also found in the SCT group (p value < 0.001) compared with the animals in the HF group.

**Conclusion:** The transplantation of hAMSCs in rats with heart failure not only decreased the level of fibrosis but also conferred significant improvement in heart performance in terms of echocardiographic and hemodynamic parameters.

## Introduction

Heart failure (HF) is a common and highly important health problem throughout the world. According to statistics, it is estimated that more than 5.2 million Americans have HF and approximately 10% to 13% of deaths are caused by HF.^1^

Mesenchymal stem cells (MSCs) are used in clinical practice for various therapeutic purposes, and their safety and possibility for use in cell therapy in the heart have been proven.^2^ The most common source of MSCs used in clinical practice is bone-marrow MSCs.^3^ Among such sources, human amniotic mesenchymal stromal cells (hAMSCs) have properties that are superior to those in other sources. The human amniotic membrane, which is an extraction source for MSCs, is readily available after each delivery and is usually discarded as medical waste. Its use does not pose risk to mother and baby, so there is no problem in its legal use. Unlike other human mesenchymal cells, hAMSCs have a high immunological tolerance and can be used as a valuable source of MSCs in transplantation and tissue repair.^3^{Scherjon, 2004 #12} Studies have shown that hAMSCs can differentiate both *in vitro *and* in vivo *in various cells such as cardiomyocytes.^4^ Furthermore, it appears that as a result of their embryonic origin, the proliferation and differentiation capacity of these cells is greater than that of the other mesenchymal cells. According to the features listed, hAMSCs have a special place in regenerative medicine. The aim of this study was to investigate the functional and histological effects of hAMSCs transplantation following *isoproterenol* (ISO)-induced global HF in male rats.

## Methods

In this research, MSCs from the human amniotic membrane were isolated and then cultured based on enzymatic methods formerly described.^5^ In short, the amnion was mechanically separated from the chorine and cut into very small pieces. The amniotic crushed pieces were digested with 0.25% trypsin (Gibco, USA) and collagens 1 (0.75 mg/mL). The cell suspension was filtered through a 70-μm Falcon cell strainer (Falcon, USA). The collected cells were seeded in 25-cm^2^Cole flasks composed of low-glucose Dulbecco’s modified eagle’s medium (DMEM-LG; Gibco, USA) plus medium supplemented with 20% fetal bovine serum (FBS; Gibco, USA). Two days later, the culture medium was replaced for the first time and only the remaining cells adhered to the bottom of the flask. These cells were gradually spindle-shaped and after 28 days, they reached 80% confluence and were passaged. The passages of the 3rd to 5th cells were used for transplantation in the cell-treated group. The pair of amniotic membrane used to isolate the MSCs was obtained from vaginal delivery.

The origin of the mesenchymal isolated cells was assessed using flow cytometry. For flow cytometry, the cell samples were prepared from passages 5. Initially, the cell suspension contained 10^3^ cells/µL by trypsin. Thereafter, 50 µL of the cell suspension and 5 µL of specific antibody FITC or PE were mixed for 30 minutes at 4 ^°^C in the dark. After fixation with 4% paraformaldehyde solution, the samples were analyzed using a flow cytometer (Partec PAS III). The following antibodies were used: CD 34–FITC (BD Pharmingen; Clone: 5E 10), CD 45–FITC, and CD 105–PE. 

For osteogenic differentiation, the isolated cells were cultured in the osteogenic induction medium containing DMEM high glucose, 10% FBS, 10^-7^ M of dexamethasone (Sigma), 10 mM of β–glycerophosphate (Merck, Darmstadt, Germany), and 0.5 mM of ascorbic acid 2-phosphate (Sigma) for 4 weeks and the media were changed every 3 days.^6^ Alizarin red staining was used to evaluate the differentiation potential of the isolated osteocytic cells. For adipogenic differentiation, the cells were incubated with the adipogenic induction medium containing DMEM, 10% FBS,10^-6^ M of dexamethasone, 0.5 mM of 3-isobutyl-1-methylxanthine (Sigma), 200 µM of indomethacin (Sigma), and 10 µg/mL of insulin (Gibco) for 4 weeks and the media were changed every 3 days. Oil Red O staining was demonstrated in evaluating the adipogenic differentiation.^7^

In order to tracking the transplanted MSCs in myocardium, Chloromethylbenzamido-1, 1´-Dioctadecyl-3,3,3´3´-Tetramethylindocarbocyanine Perchlorate (CM-DiI) was used as a marker. The stock solution of lipophilic tracers CM-DiI (Cell Tracker CM-DiI; Invitrogen, USA) was prepared according to the manufacturer’s instructions. The final concentration of CM-DiI stock was made up to a concentration of 1 mg/mL in dimethyl sulfoxide (DMSO; Sigma). Immediately before labeling, the stock solution was diluted with Dulbecco’s phosphate-buffered saline (D-PBS).

The hAMSCs were harvested 28 days after the last ISO injection, and MSCs staining at 1-µM (molecular weight CM-DiI is close to 1000; therefore, 1 µM~1 µg/mL) concentrations was performed for 5 minutes at 37 ^°^C and finally for 15 minutes at 4 ^°^C, with occasional mixing to improve *staining* levels according to the manufacturer’s standard protocol.^8^ After being labeled, the MSCs were washed with PBS and resuspended in 150 µL of a fresh medium and injected into the myocardium. In this study, 28 days after the transplantation of hAMSCs, the animals in the SCT group (n = 7) were sacrificed, and paraffin blocks were prepared from the heart. Then, slides with a thickness of 5 µm were prepared and analyzed using a microscope (Olympus, Tokyo, Japan).

The protocol was approved by the institutional Care and Use Committee of Iran University of Medical Sciences (Tehran, Iran). The experiments were performed on 35 male Wistar rats (180-200 g), which were housed in controlled environment conditions (22 ± 2 ^°^C; light – dark cycle 7 am – 7 pm). The rats were allowed to access water and standard laboratory food *ad libitum*. ISO-induced global HF was effected as described in a previous study.^9^ In summary, ISO (170 mg/kg dissolved in 0.5 mL of saline, Sigma, USA) was subcutaneously injected into the rats every day for 4 sequential days. Twenty-eight days after the last injection of ISO, the establishment of HF was confirmed with echocardiography through ejection fraction percentage (EF, %) measurement (EF < 70%), and the animals with EF > 70% were not used in the intervention groups. The animals were divided into 5 groups (n = 7, each group). Four weeks post ISO injection, failure confirmation was done by echocardiography. The hAMSCs (3 × 10^6^) were dissolved in 150 µL of a culture medium and injected into 4 points along the left anterior descending artery (LAD) path (stem cell transplantation [SCT] group). The culture medium without hAMSCs in the same volume was also injected accordingly (CM group). Four points along the LAD path were also punctured post failure induction without cell injection (sham group). The control (con) group, animals received no intervention and in a group called heart failure (HF) group were injected only ISO and surgical intervention was not performed. In all the groups studied (n = 7 from each group), the rats were evaluated at the beginning of the study and a similar echocardiogram was performed again 4 and 8 weeks after the last ISO injection. The echocardiograms were done following mild anesthesia (induced with ketamine [80 mg/kg] and xylazine [8 mg/kg], intraperitoneally) and carried out under continuous electrocardiogram (ECG) monitoring with an echocardiographic system equipped with a 6–12 MHz linear transducer (GE Voluson 730 Pro, Kretztechnik Company, Austria).  The echocardiographic investigations were carried out by a researcher who was unaware of the treatment protocol. First, 2-dimensional (B-mode) images were obtained in the middle section of the parasternal short-axis view of the left ventricle (LV). At the level of the papillary muscles, M-mode tracings from the parasternal long-axis view were used to measure the echocardiographic parameters: LV end-diastolic dimension (LVD_d_, cm), LV end-systolic dimension ([LVD_s_], cm), intraventricular septal width in diastole ([IVS_d_], cm), intraventricular septal width in systole (IVS_s_, cm), LV end-diastolic posterior wall thickness (LVPWT_d_, cm), and LV end-systolic posterior wall thickness (LVPWT_s_, cm). Fractional shortening (FS, %) was calculated as (LVD_d_-LVD_s_)**/**LVD_d_] × 100. Additionally, EF (%) was calculated as [(LVD_d_^3^-LVD_s_^3^)**/**LVD_d_^3^] × 100. All the parameters were measured from at least 3 successive cardiac cycles and averaged.

The hemodynamic parameters in all the groups studied (n = 7 from each group) were evaluated at 8 weeks after the last ISO injection, following echocardiography. The rats were anesthetized with thiopental (60 mg/kg) and under continuous ECG monitoring, a polyethylene tube (PE50, 0.58 mm ID **×** 0.96 mm OD, Portex, England) filled with heparin saline (500 U/mL) was inserted into the carotid artery. This catheter was attached to the Power Lab data acquisition system via a pressure transducer (AD Instrument Pty Ltd, Mountain View, CA, USA) and thereby systolic blood pressure (mmHg) and diastolic blood pressure (mmHg) were constantly monitored and recorded on a computer throughout the research. Thereafter, the catheter was carefully pushed forward into the LV. LV systolic pressure (LVSP, mmHg), LV end-diastolic pressure (LVEDP, mmHg), maximum rate of LV pressure rise (+dP/dt, mmHg/s; contractility), and maximum rate of LV pressure decline (-dP/dt, mmHg/s, relaxation) were monitored and recorded. Also, rate pressure product (beats × mmHg/min) was obtained by multiplying heart rate in LV developed pressure (LVDP). LVDP was the result of the pressure difference of the systolic and diastolic pressures of the LV. Upon the completion of the hemodynamic evaluation, the animals were sacrificed and their heart was immediately removed by cutting the large blood vessels connected to it.

For histological studies, 8 weeks after the last ISO injection, at the end of the hemodynamic parameter measurements, the rats (n = 7 from each group) were sacrificed and cut into 3 transverse segments parallel to the atrioventricular groove. After the preparation of the paraffin blocks of the heart tissue, slices were prepared with a thickness of 5 µm. Masson’s trichrome and hematoxylin and eosin (H & E) staining were performed to study the histological changes. 

Masson’s trichrome staining was conducted to evaluate interstitial fibrosis in the myocardium. The surface area of the fibrosis of the cardiac muscle was calculated as follows: the transverse sections were randomly obtained from 2 levels (basal and apical), and 10 randomly chosen fields for each section (n = 20 per animal) were analyzed. Then, for each field at 200 × magnification, photography was done using a digital photography camera and the images were stored in a computer. Image J, version 1.48, (US National Institutes of Health, Bethesda, MD, USA) was used to measure the amount of fibrosis in each section. The percentage of fibrotic tissue infiltration in the LV was calculated as follows^10^: (Fibrotic tissue area)**/**(fibrotic tissue area + myocyte area) × 100%. 

The statistical analyses were performed with Statistical Package for the Social Sciences (SPSS) (version 21.0; SPSS, Chicago, IL). The data are presented as means ± SDs. The independent sample *t*-test was used for the comparison of the echocardiographic indices between the control and HF groups to confirm HF. The one-way ANOVA and Bonferroni post hoc test were utilized to compare the echocardiographic and hemodynamic indices between the groups. A p value < 0.05 was considered statistically significant.

## Results

The rats were carefully monitored throughout the study period in terms of the mortality rate. Fifty-one rats were used at the beginning of the study. Of these rats, 7 were randomly selected as the control group (intact animals). Twelve out of the remaining 44 animals died in the first 24 hours post ISO injection. Four rats also died in the second post ISO injection. Finally, the remaining 28 rats were randomly placed in equally numbered groups. No death was found in the control group during the investigation.

**Figure 1:**
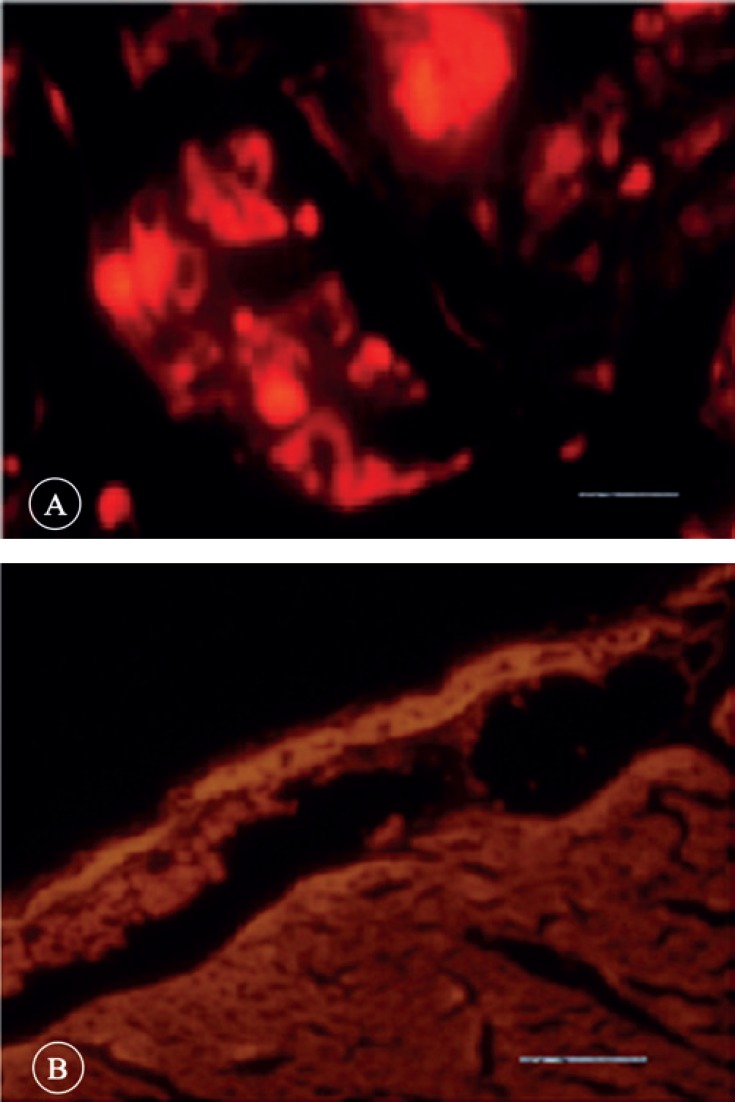
Two fluorescence microscopy views of a region in the myocardial

Four weeks after transplantation, the cells labeled with a fluorescent dye (CM-DiI) showed the existence of transplanted hAMSCs in the myocardium of the animals from the SCT group (Figure 1). 

**Figure 2:**
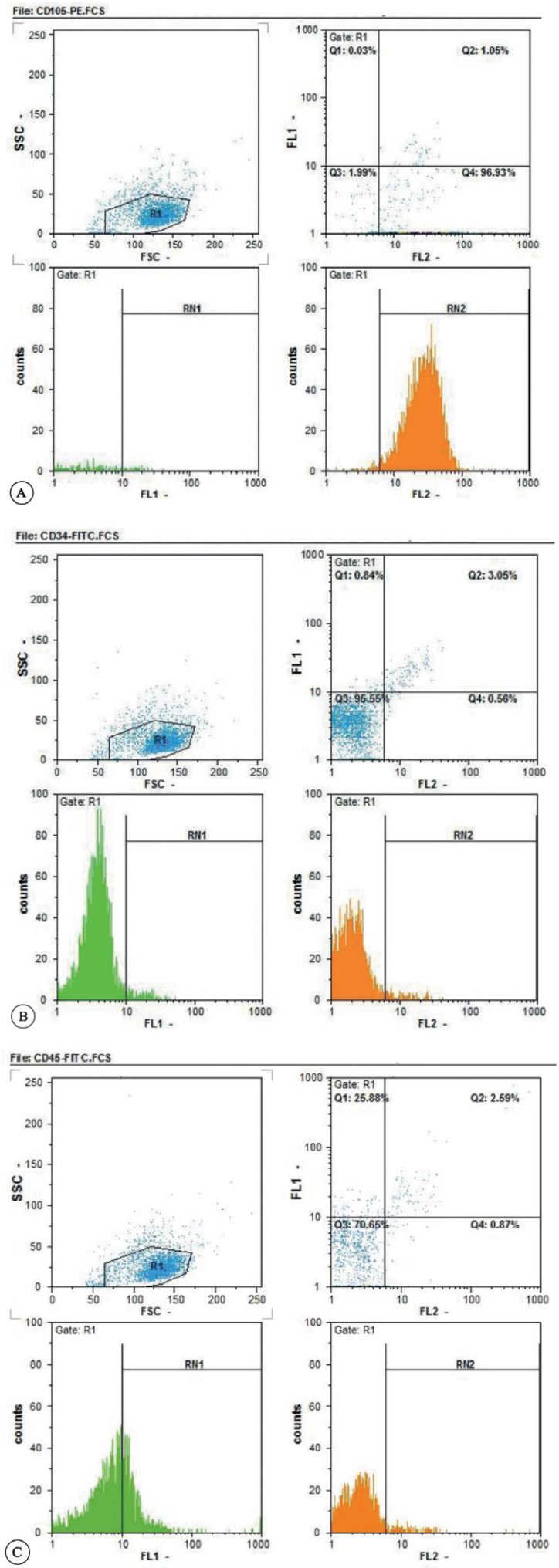
Immunophenotypic characterization of the amniotic membrane-derived stem cells. These cells expressed mesenchymal stem cell marker CD105 (A), whereas they were negative for CD34 (B) and CD 45 (C).

The flow cytometry results showed that the isolated cells expressed the MSC marker CD105 but did not express the hematopoietic progenitor marker CD34 and the pan-leukocyte marker CD 45 (Figure 2).

**Figure 3:**
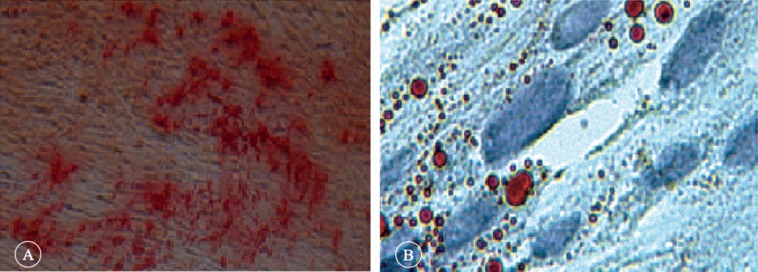
The microscopic images differentiation human amniotic membrane-derived mesenchymal cell into osteocytes and adipocytes

Three days after cell isolation, the mesenchymal cells were cultured in the adipogenic induction medium, and lipid droplets were observed in some cells. With time, these droplets gradually increased in cells and after 21 days of culture, the lipid droplets were observed to be reddish in color with Oil Red O staining (Figure 3A). After 21 days of culture, with alizarin red staining, cell mass in terms of mineral matrix secretion was evaluated and the result showed red cell mass, which implied that the test was positive (Figure 3B).

Four weeks after the last ISO injection, LVD_s _(p value < 0.001), LVD_d_ (p value = 0.009), and IVS_s_ (p value = 0.012) were all increased, while EF (p value < 0.001) and FS (p value < 0.001) decreased compared with the values in the control animals. This was indicative of HF development. 

**Figure 4:**
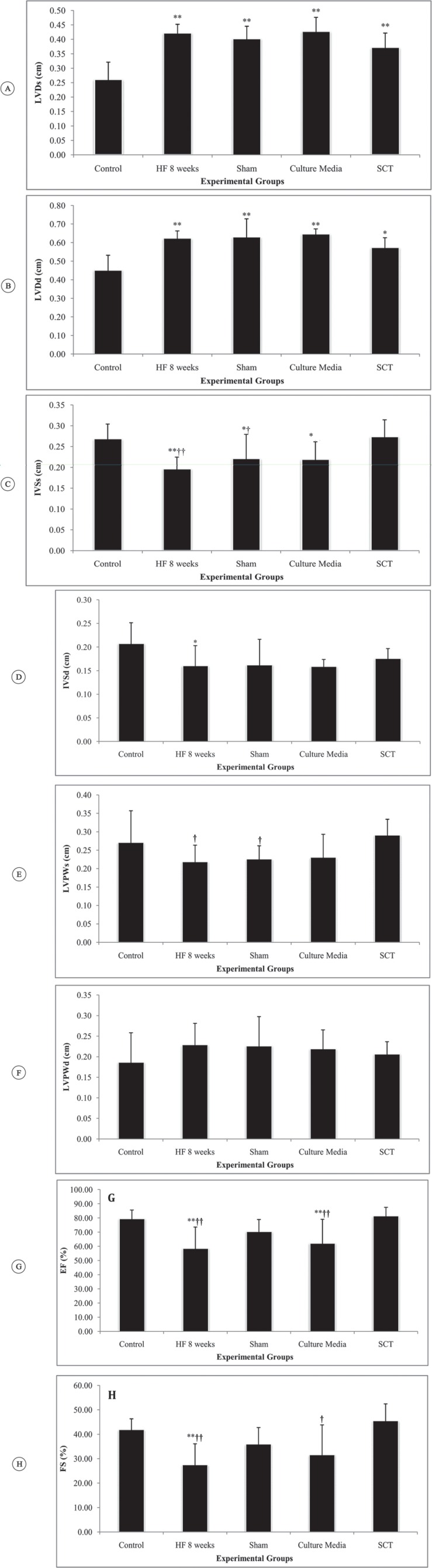
Effects of human amniotic mesenchymal stromal cells (hAMSCs) transplantation on left ventricular (LV) echocardiographic parameters in the studied animals (each group n=7). A) LVDs (Left ventricular end-systolic dimension). B) LVDd (LV end-diastolic dimension). C) IVSs (Intraventricular septal width in systole). D) IVSd (Intraventricular septal width in diastole). E) LVPWTs (LV end-systolic posterior wall thickness). F) LVPWTd (LV end-diastolic posterior wall thickness). G) EF (Ejection fraction). H) FS (Fractional shortening). Con, control group; HF 8 weeks, rats with heart failure in eight weeks after injection of isoproterenol.

Eight weeks after the last ISO injection, LVD_s_ (df: 33; F = 21.10; p value < 0.001) and LVD_d_ (df: 33; F = 15.26; p value < 0.001) showed changes in all different animals. These parameters were significantly higher in the HF group than in the control group) p value < 0.001). Although hAMSCs transplantation decreased LVD_s _(p value = 0.078) and LVD_d_ (p value = 0.459) compared to the values in the HF group, the effect was not statistically significant. The transplantation of hAMSC_s_ in the SCT group failed to bring LVD_s _(p value < 0.001) and LVD_d _(p value = 0.001) to the values in the control group (Figure 4A & Figure 4B).

Eight weeks’ follow-up showed a significant difference in IVS_s_ (df: 33; F = 7.86; p value < 0.001) and IVS_d _(df: 33; F = 2.7; p value = 0.041) among the studied groups. IVS_s_ (p value = 0.001) and IVS_d_ (p value = 0.049) in the HF group were significantly lower than those in the control animals. The transplantation of hAMSCs increased IVS_s_ diameter compared to that in the HF group (p value < 0.001). There was no significant difference between the SCT group and the control group (p value = 0.647). However, this therapeutic strategy did not affect IVS_d_ (p value = 0.015) (Figure 4C & Figure 4D).

LVPW_s_ was statistically different between the groups (df: 33; F = 3.50; p value = 0.014), but LVPW_d_ did not change (df: 33; F = 1.0; p value = 0.422). LVPW_s_ was thicker in the SCT group than in the HF group (p value = 0.024) and the sham group (p value = 0.035) (Figure 4E & Figure 4F). Furthermore, EF (df: 33; F = 8.68; p value < 0.001) and FS (df: 33; F = 9.68; p value < 0.001) were significantly decreased 8 weeks after HF induction. However, hAMSCs transplantation provoked a significant increase in EF (p value < 0.001) and FS (p value < 0.001) in comparison with those in the HF group (Figure 4G & Figure 4H). There was no significant difference between the SCT group and the control group. (p value = 1.000) (Table 1). 

A) Chloromethylbenzamido-1,1´-Dioctadecyl-3,3,3´3´Tetramethylindocarbocyanine Perchlorate (CM-DiI) labeled mesenchymal stem cells (MSCs) can be seen as bright red color spots in the myocardium. B) View from the same spot in the myocardium that did not receive stem cell transplant.

Magnification × 200, Scale bar = 100 nm

A) Osteogenic differentiation was evidenced by the formation of extracellular calcium deposits (alizarin red staining positive) which can be seen as red regions. B) Adipogenic differentiation was confirmed by the formation of intracellular lipid droplet (Oil Red O staining positive) these droplets can be seen as red regions.

Magnification × 200

Sham, Sham operating group; CM, Culture media group; SCT, Stem cell transplantation group.

Data are presented as means±SD

*: significant difference vs. control, *p value < 0.05, **p value < 0.001

†: significant difference vs. SCT, † p value < 0.05, ††: p value < 0.001

**Table 1:** Echocardiographic findings in the experimental groups*

	Studied Groups						P value
	Control	HF 4 weeks	HF 8 weeks	Sham	Culture Media	SCT	
LVDs (cm)	0.26±0.06	0.40±0.07	0.42±0.03	0.40±0.04	0.43±0.05	0.37±0.05	< 0.001
LVDd (cm)	0.45±0.08	0.59±0.11	0.62±0.04	0.63±0.10	0.65±0.03	0.57±0.05	< 0.001
IVSs (cm)	0.27±0.04	0.21±0.04	0.20±0.03	0.22±0.06	0.22±0.04	0.27±0.04	< 0.001
IVSd (cm)	0.21±0.04	0.16±0.04	0.16±0.04	0.16±0.05	0.16±0.01	0.18±0.02	0.041
LVPWs (cm)	0.27±0.09	0.21±0.04	0.22±0.05	0.23±0.04	0.23±0.06	0.29±0.04	0.014
LVPWd (cm)	0.19±0.07	0.19±0.04	0.23±0.05	0.23±0.07	0.22±0.05	0.21±0.03	0.422
EF (%)	79.67±5.90	65.73±9.51	58.73±14.85	70.63±8.25	62.25±16.79	81.45±6.06	< 0.001
FS (%)	41.94±4.41	31.87±6.55	27.53±8.58	36.00±6.76	31.63±12.22	45.55±6.91	< 0.001

HF 4 weeks, Rats with heart failure in four weeks after injection of isoproterenol; HF 8 weeks, Rats with heart failure in eight weeks after injection of isoproterenol;

SCT, Stem cell transplantation; LVDs, Left ventricular internal dimension in systole; LVDd, Left interventricular internal dimension in diastole; IVSs, Interventricular septal thickness in systole; IVSd, Interventricular septal thickness in diastole; LVPWs, Left ventricular posterior wall in systole; LVPWd, Left ventricular posterior wall in diastole; EF, Ejection fraction; FS, Fraction shortening.

* Data are presented as mean±SD

**Table 2:** Hemodynamic assessment in the experimental groups*

	Studied Groups					P value
	Control	HF 8 weeks	Sham	Culture Media	SCT	
HR (beat/min)	379.35±14.53	382.34±27.05	360.29±22.20	365.51±27.75	378.07±37.52	0.580
SBP (mmHg)	130.02±12.98	119.76±10.94	112.94±15.20	109.02±17.10	126.77±10.09	0.074
DBP (mmHg)	110.76±7.66	88.26±10.13	94.18±12.70	91.71±14.02	101.33±13.67	0.031
MAP (mmHg)	117.16±9.26	98.70±9.58	100.39±13.43	97.47±14.67	109.87±11.94	0.045
Max dP/dt (mmHg/s)	4517.50±377.78	3046.60±215.62	3016.00±344.09	2938.60±278.91	3637.20±181.84	< 0.001
Min dP/dt (mmHg/s)	-3331.67±368.86	-2262.00±256.68	-2128.20±143.60	-2196.80±112.13	-2541.72±374.79	< 0.001
LVSP mmHg	8.36±2.49	28.88±10.43	29.39±6.59	28.29±6.73	14.40±10.98	0.003
LVEDP mmHg	115.91±17.94	84.09±10.82	88.69±8.52	91.84±11.41	109.42±16.65	< 0.001
RPP (beat/min × mmHg)	41671.20±7264.27	20975.00±5639.48	21413.67±3449.53	21231.40±3163.38	38270.17±8303.96	< 0.001

HF 8 weeks, Rats with heart failure in eight weeks after injection of isoproterenol.

HF, Heart failure; SCT, Stem cell transplantation; HR, Heart rate ; SBP, Systolic blood pressure; DBP, Diastolic blood pressure; MAP, Mean arterial pressure; LVSP, Left ventricular systolic pressure; LVEDP, Left ventricular end-diastolic pressure; Max dP/dt, Maximum rate of left ventricular pressure rise; Min dP/dt, Maximum rate of left ventricular pressure decline; RPP, Rate pressure product

* Data are presented as mean±SD

Our results did not show significant differences in heart rate (df: 33; F = 0.72; p value = 0.580) and systolic blood pressure (df: 33; F = 1.46; p value = 0.074) between the groups, while mean diastolic blood pressure (df: 33; F = 3.21; p value = 0.031), mean arterial pressure (df: 33; F = 2.88; p value = 0.045), max dP/dt (df: 33; F = 29.17; p value < 0.001), min dP/dt (df: 33; F = 17.87; p value < 0.001), LVSP (df: 33; F = 5.60; p value = 0.003), LVEDP (df: 33; F = 7.61; p value < 0.001), and rate pressure product (df: 33; F 16.07; p value < 0.001) were strikingly different between the studied animals (Table 2).

**Figure 5:**
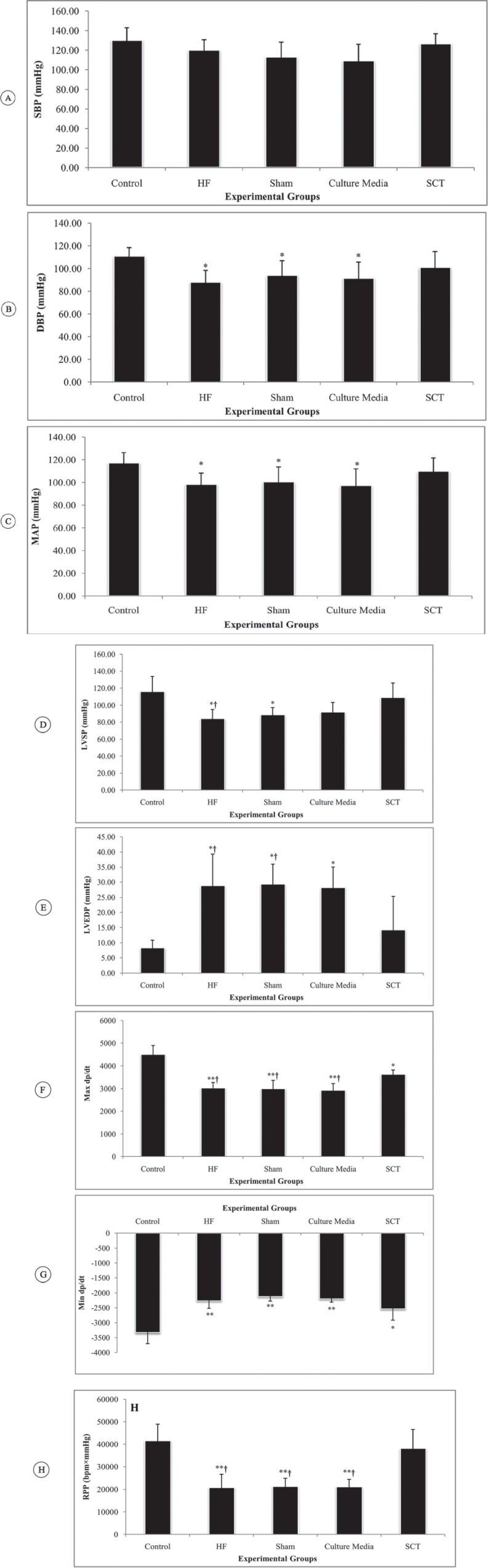
Effects of human amniotic mesenchymal stromal cells (hAMSCs) transplantation on left ventricular (LV) hemodynamic parameters in the studied animals (each group n = 7).

As was expected, HF induction caused a significant decrease in diastolic blood pressure (p value = 0.044), mean arterial pressure (p value = 0.046), LVSP (p value = 0.011), max dP/dt (p value < 0.001), and rate pressure product (p value < 0.001) but provoked a marked increase in LVEDP (p value = 0.007) and min dP/dt (p value < 0.001) compared to the same values in the control animals. The transplantation of hAMSCs reversed these complications. There was no significant difference between the SCT group and the control group in diastolic blood pressure (p value = 0.180), mean arterial pressure (p value = 0.288), LVSP (p value = 0.106), rate pressure product (p value = 0.501), and LVEDP (p value = 0.982) (Figure 5). However, mean max dP/dt (p value = 0.001) and min dP/dt (p value = 0.001) were significantly higher than those in the control group.

**Figure 6:**
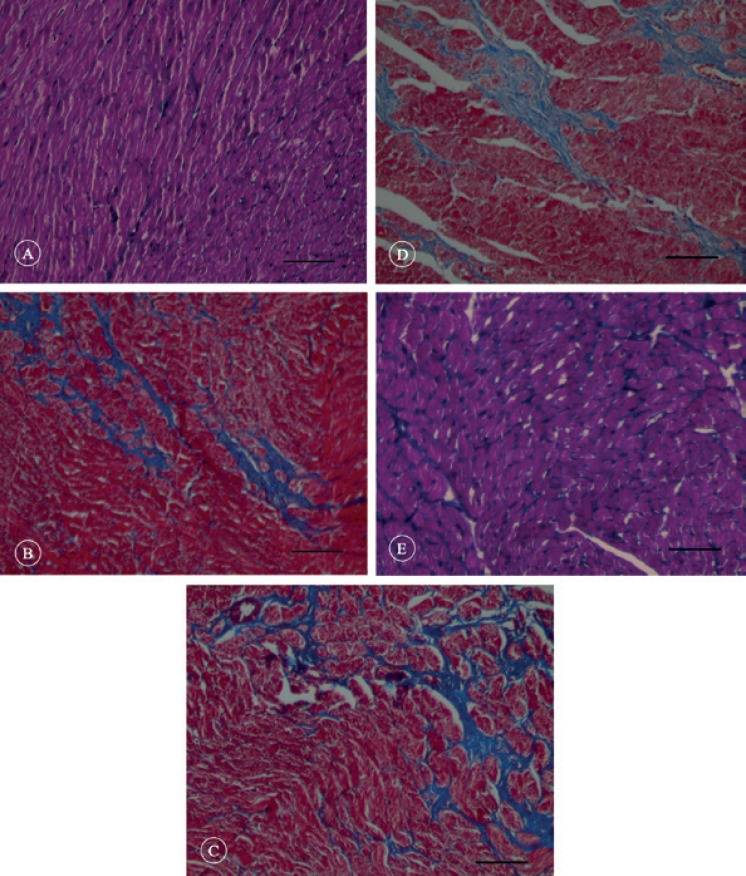
Cross sections of left ventricular myocardial tissue stained with trichrome mission. In these images fibrous areas appear turquoise blue in color.

**Figure 7:**
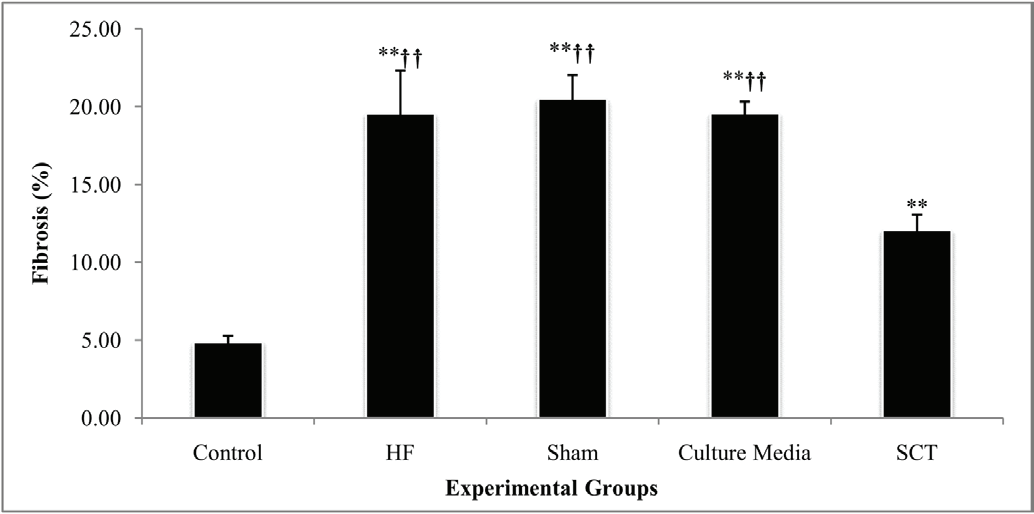
Effects of human amniotic mesenchymal stromal cells (hAMSCs) transplantation on myocardial fibrosis fraction in the studied animals (each group n = 7).

**Figure 8:**
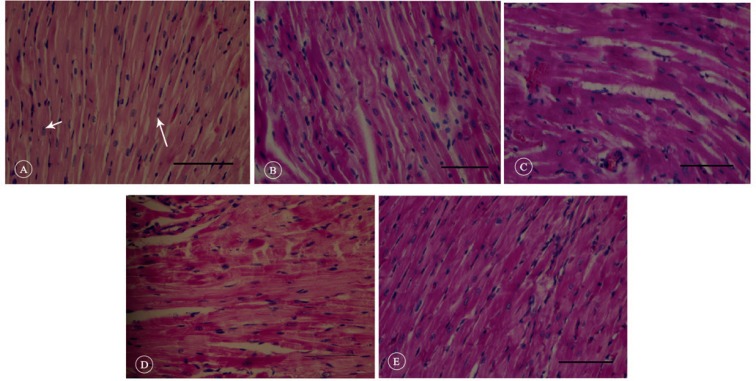
Representative hematoxylin-eosin stained myocardial sections in the studied animals

Our quantitative analysis showed that fibrosis tissue percentage significantly increased in the HF group compared with that in the control group (19.56 ± 2.76% vs. 4.85 ± 0.43%; p value < 0.001). The implantation of hAMSCs significantly reduced fibrosis formation (Figure 6). However, fibrosis percentage was significantly higher in the SCT group than in the control animals (12.07 ± 1.12% vs. 4.85 ± 0.43%; p value < 0.001) (Figure 7). In addition, H&E staining showed that the myocardial tissues changed and lost their integrity and order after HF induction. The space between the cells and cardiomyocytes was also increased, which made them lose their natural structure. In the SCT group, there was repair of the damage, approximating the myocardial tissue to that of the healthy rats. Also, the order and integrity of cardiomyocytes were re-established (Figure 8).

A) SBP (systolic blood pressure), B) DBP (diastolic blood pressure), C) MAP (mean arterial pressure), D) LVSP (left ventricular systolic pressure), E) LVEDP (left ventricular end-diastolic pressure), F) + dP/dt (maximum rate of left ventricular pressure rise), G) -dP/dt (Maximum rate of left ventricular pressure decline), and H) RPP (rate pressure product).

Control, control group; HF, Heart failure group; Sham, Sham operating group; CM, Culture media group; SCT, Stem cell transplantation group

Data are presented as means±SD.

*significant difference vs. control, *p value < 0.05, **p value < 0.001

†significant difference vs. SCT, †p value < 0.05, ††p value < 0.001

A) Control group, B) Heart failure group, C) Culture media group, D) Sham operating group, and E) Stem cell transplantation group

Magnification × 200

Scale bar = 100 nm

Control, control group; HF, Heart failure group; Sham, Sham operating group; CM, Culture media group; SCT, Stem cell transplantation group

*significant difference vs. control, *p value < 0.05, **p value < 0.001

†significant difference vs. SCT, †p value < 0.05, ††p value < 0.001

Great flash shows cardiomyocytes with normal nucleus and small arrow shows cardiomyocytes with nucleus pyknotic.

A) Control group, B) Heart failure group, C) Culture media group, D) Sham operating group, and E) Stem cell transplantation group

Magnification × 400

Scale bar = 100 nm

## Discussion

In the near future, stem cell therapy may become an alternative treatment for cardiac failure. There are various methods for creating models of HF in laboratory animals.^11^ ISO-induced HF is a standard method in which extensive myocardial damage gradually progresses (it is, therefore, a time-dependent process),^9^ while the vascular system remains intact in the heart. It is almost like the natural process that occurs *in vivo* during HF.^12^ In the present study, we used this model of HF induced by ISO.

Our results confirmed the findings of previous studies insofar as-in comparison with the control group-HF increased heart dimensions (increase in LVD_s_ and LVD_d_) and reduced heart function (decrease in EF).^13^ Increased LVEDP in the HF group may have been due to impaired LV pumping function and inadequate emptying, which then might have provoked ventricular dilation and blood retention. Our data analysis showed that hAMSCs transplantation decreased LVEDP. This suggests that cell therapy prevents ventricular dilation and collection of blood in the LV. Our evaluation of the hemodynamic parameters indicated changes in the index contraction and relaxation of the LV in HF. Indeed during HF, collagen due to increased levels of matrix metalloproteinases (MMPs) and interstitial fibrosis collagen with cross bridges are poorly made, which in turn may lead to ventricular dilation and myocardial dysfunction.^14^

In the cell-treated group, the heart dimensions were decreased, and EF and FS were significantly increased. These results chime in with those in a study by Li et al.^9^ Studies have shown that MSCs transplantation can inhibit the expression of MMPs and collagen, which is capable of reducing the dimensions of the heart and causing dilation. Similar to the investigation by Li et al.,^9^ we demonstrated that IVS_s_ and IVS_d_ were reduced in HF and improved with stem cell transplantation. The decrease in heart dimensions improved the function of the ventricles in smaller dimensions,^15^ like what which seen in the natural state and prevented the cardiomyocytes from stretching too much, thereby conferring better contractile power.^16^

In the present study, hemodynamic parameters were also measured to assess ventricular function.^12^ The ventricular remodeling that occurs during HF may cause cardiac dysfunction in both systole and diastole.^9^ A reduction in systolic blood pressure, diastolic blood pressure, and LVSP following HF induction can be caused by the death of cardiomyocytes, myocardial necrosis, and myocardial stunning. Increased systolic and diastolic pressures following hAMSCs transplantation may be partly due to the conversion of the transplanted MSCs into cardiomyocytes^4^ and myocardial stem cells paracrine factors (such as hepatocyte growth factor) with trophic and cardioprotective properties.              17^, ^^18^ 

The results of the current study showed the effect of hAMSCs transplantation on reducing the amount of fibrosis in the myocardium, as compared to other cells used in previous studies which are more. For example, in the study by Li et al.,^9^ the fibrosis content of the myocardium in rats with HF decreased from 11.3% to 9.8% after the transplantation of bone-marrow MSCs. In contrast in the present study, the extent of fibrosis in the myocardium of the rats with HF decreased from 19.5% to 12.0% after hAMSCs transplantation. In their study, Li et al.^9^ could not find evidence of the differentiation of bone-marrow-derived MSCs into cardiomyocytes. Therefore, a reduction in myocardial fibrosis can cause the release of MSCs paracrine factors. Studies have confirmed that hAMSCs could be transdifferentiated into cardiomyocytes *in vitro* and *in vivo*, without requiring any epigenetic factor or gene transfer.^17^ Thus, it seems that part of the reduction in myocardial fibrosis and cardiac repair is related to the homing and differentiation of stem cells into cardiomyocytes. ^12^^, ^^19^ It should also be noted that autocrine and paracrine products released by the transplanted cells are involved in reducing the damage caused by HF in the myocardium.^20^^, ^^21^ Accordingly, it seems that statistically the effect of hAMSCs on fibrous reduction is more than bone marrow-derived mesenchymal stem cells. Because hAMSCs act in both ways, they differentiate into cardiomyocytes and bring about the delivery of autocrine and paracrine factors.

Increased collagen content and fibrosis in the interstitial space have been seen in many cases of myocardial hypertrophy such as which occurs during HF, and they trigger a variety of changes in ventricular pressures.^10^^, ^^22^ Hence, it seems that one of the ways in which MSCs transplantation can improve cardiac function is by reducing myocardial fibrosis.

Lifestyle change, improved nutrition, and pharmacological therapy with medications such as ivabradine^23^ and LCZ696^24^ have little effects on heart performance in cardiac failure states and can only slow its progression, without any compensatory influence on cell loss.^25^^, ^^26^ The transplantation of hAMSCs allows non-functional cardiomyocytes and scar tissues to be replaced with healthy cells with normal function and can revert the side effects of HF for good in comparison with transient palliative modalities.

## Conclusion

This study recorded significant improvement in cardiac function and reduction in cardiac fibrosis in the stem cell transplantation group when compared with the heart failure, sham and culture medium groups. The results of this study suggest that human amniotic mesenchymal stromal cell transplantation has cardioprotective effects on heart failure. Evidence for this cardioprotective effect comes from the results of echocardiographic studies, measurement of hemodynamic parameters, and histological findings. Therefore, it seems that human amniotic mesenchymal stromal cell transplantation has the potential to be used for the treatment of heart failure.

## Notes:

***This paper should be cited as:*** Razavi Tousi SMT, Faghihi M, Nobakht M, Molazem M, Kalantari E, Darbandi Azar A, Aboutaleb N. Improvement of Cardiac Heart Failure by Human Amniotic Mesenchymal Stromal Cell Transplantation in Rats. J Teh Univ Heart Ctr 2016;11(3):123-138.

## References

[1] Rosamond W, Flegal K, Friday G, Furie K, Go A, Greenlund K, Haase N, Ho M, Howard V, Kissela B, Kittner S, Lloyd-Jones D, McDermott M, Meigs J, Moy C, Nichol G, O'Donnell CJ, Roger V, Rumsfeld J, Sorlie P, Steinberger J, Thom T, Wasserthiel-Smoller S, Hong Y, American Heart Association Statistics Committee and Stroke Statistics Subcommittee (2007). Heart disease and stroke statistics--2007 update: a report from the American Heart Association Statistics Committee and Stroke Statistics Subcommittee. Circulation.

[2] Chen SL, Fang WW, Ye F, Liu YH, Qian J, Shan SJ, Zhang JJ, Chunhua RZ, Liao LM, Lin S, Sun JP (2004). Effect on left ventricular function of intracoronary transplantation of autologous bone marrow mesenchymal stem cell in patients with acute myocardial infarction. Am J Cardiol.

[3] In 't Anker PS, Scherjon SA, Kleijburg-van der Keur C, de Groot-Swings GM, Claas FH, Fibbe WE, Kanhai HH (2004). Isolation of mesenchymal stem cells of fetal or maternal origin from human placenta. Stem Cells.

[4] Tsuji H, Miyoshi S, Ikegami Y, Hida N, Asada H, Togashi I, Suzuki J, Satake M, Nakamizo H, Tanaka M, Mori T, Segawa K, Nishiyama N, Inoue J, Makino H, Miyado K, Ogawa S, Yoshimura Y, Umezawa A (2010). Xenografted human amniotic membrane-derived mesenchymal stem cells are immunologically tolerated and transdifferentiated into cardiomyocytes. Circ Res.

[5] Zhang D, Jiang M, Miao D (2011). Transplanted human amniotic membrane-derived mesenchymal stem cells ameliorate carbon tetrachloride-induced liver cirrhosis in mouse. PLoS One.

[6] Sudo K, Kanno M, Miharada K, Ogawa S, Hiroyama T, Saijo K, Nakamura Y (2007). Mesenchymal progenitors able to differentiate into osteogenic, chondrogenic, and/or adipogenic cells in vitro are present in most primary fibroblast-like cell populations. Stem Cells.

[7] Alviano F, Fossati V, Marchionni C, Arpinati M, Bonsi L, Franchina M, Lanzoni G, Cantoni S, Cavallini C, Bianchi F, Tazzari PL, Pasquinelli G, Foroni L, Ventura C, Grossi A, Bagnara GP (2007). Term Amniotic membrane is a high throughput source for multipotent Mesenchymal Stem Cells with the ability to differentiate into endothelial cells in vitro. BMC Dev Biol.

[8] Dai WF, Hale SL, Martin BJ, Kuang JQ, Dow JS, Wold LE, Kloner RA (2005). Allogeneic mesenchymal stem cell transplantation in postinfarcted rat myocardium: short- and long-term effects. Circulation.

[9] Li L, Zhang Y, Li Y, Yu B, Xu Y, Zhao S, Guan Z (2008). Mesenchymal stem cell transplantation attenuates cardiac fibrosis associated with isoproterenol-induced global heart failure. Transpl Int.

[10] Kang NN, Fu L, Xu J, Han Y, Cao JX, Sun JF, Zheng M (2012). Testosterone improves cardiac function and alters angiotensin II receptors in isoproterenol-induced heart failure. Arch Cardiovasc Dis.

[11] Carll AP, Willis MS, Lust RM, Costa DL, Farraj AK (2011). Merits of non-invasive rat models of left ventricular heart failure. Cardiovasc Toxicol.

[12] Grimm D, Elsner D, Schunkert H, Pfeifer M, Griese D, Bruckschlegel G, Muders F, Riegger GA, Kromer EP (1998). Development of heart failure following isoproterenol administration in the rat: role of the renin-angiotensin system. Cardiovasc Res.

[13] Miki K, Uenaka H, Saito A, Miyagawa S, Sakaguchi T, Higuchi T, Shimizu T, Okano T, Yamanaka S, Sawa Y (2012). Bioengineered myocardium derived from induced pluripotent stem cells improves cardiac function and attenuates cardiac remodeling following chronic myocardial infarction in rats. Stem Cells Transl Med.

[14] Vanhoutte D, Schellings M, Pinto Y, Heymans S (2006). Relevance of matrix metalloproteinases and their inhibitors after myocardial infarction: a temporal and spatial window. Cardiovasc Res.

[15] Tomita S, Li RK, Weisel RD, Mickle DA, Kim EJ, Sakai T, Jia ZQ (1999). Autologous transplantation of bone marrow cells improves damaged heart function. Circulation.

[16] Li RK, Jia ZQ, Weisel RD, Mickle DA, Zhang J, Mohabeer MK, Rao V, Ivanov J (1996). Cardiomyocyte transplantation improves heart function. Ann Thorac Surg.

[17] Tse HF, Siu CW, Zhu SG, Songyan L, Zhang QY, Lai WH, Kwong YL, Nicholls J, Lau CP (2007). Paracrine effects of direct intramyocardial implantation of bone marrow derived cells to enhance neovascularization in chronic ischaemic myocardium. Eur J Heart Fail.

[18] Uemura R, Xu M, Ahmad N, Ashraf M (2006). Bone marrow stem cells prevent left ventricular remodeling of ischemic heart through paracrine signaling. Circ Res.

[19] Zhao Y, Li T, Wei X, Bianchi G, Hu J, Sanchez PG, Xu K, Zhang P, Pittenger MF, Wu ZJ, Griffith BP (2012). Mesenchymal stem cell transplantation improves regional cardiac remodeling following ovine infarction. Stem Cells Transl Med.

[20] Caspari PG, Newcomb M, Gibson K, Harris P (1977). Collagen in the normal and hypertrophied human ventricle. Cardiovasc Res.

[21] Weber KT, Janicki JS, Pick R, Abrahams C, Shroff SG, Bashey RI, Chen RM (1987). Collagen in the hypertrophied, pressure-overloaded myocardium. Circulation.

[22] Lim KH, Ko D, Kim JH (2013). Cardioprotective potential of Korean Red Ginseng extract on isoproterenol-induced cardiac injury in rats. J Ginseng Res.

[23] Markandeya YS, Phelan LJ, Woon MT, Keefe AM, Reynolds CR, August BK, Hacker TA, Roth DM, Patel HH, Balijepalli RC (2015). Caveolin-3 Overexpression Attenuates Cardiac Hypertrophy via Inhibition of T-type Ca2+ Current Modulated by Protein Kinase Cα in Cardiomyocytes. J Biol Chem.

[24] Szema AM, Dang S, Li JC (2015). Emerging Novel Therapies for Heart Failure. Clin Med Insights Cardiol.

[25] Müller-Ehmsen J, Whittaker P, Kloner RA, Dow JS, Sakoda T, Long TI, Laird PW, Kedes L (2002). Survival and development of neonatal rat cardiomyocytes transplanted into adult myocardium. J Mol Cell Cardiol.

[26] Fuchs S, Satler LF, Kornowski R, Okubagzi P, Weisz G, Baffour R, Waksman R, Weissman NJ, Cerqueira M, Leon MB, Epstein SE (2003). Catheter-based autologous bone marrow myocardial injection in no-option patients with advanced coronary artery disease: a feasibility study. J Am Coll Cardiol.

